# Trends in good laboratory practice studies submitted for the marketing authorization of pharmaceuticals in Japan

**DOI:** 10.3389/fphar.2025.1591433

**Published:** 2025-07-04

**Authors:** Yusuke Oku, Hitoshi Someya

**Affiliations:** Pharmaceuticals and Medical Devices Agency, Tokyo, Japan

**Keywords:** good laboratory practice (GLP), non-clinical safety study, pharmaceuticals, regenerative medical products, Organisation for Economic Cooperation and Development (OECD), mutual acceptance of data (MAD), non-MAD country

## Abstract

Pharmaceuticals and Medical Devices Agencies (PMDA) receives the non-clinical safety studies following Good Laboratory Practice (GLP) for the marketing authorization of medical products. Here we analyzed the trends of GLP-compliant non-clinical studies (GLP studies) submitted to PMDA from FY2017 to FY2023. Geographical analysis of the origin of studies revealed that the share of Japanese studies slightly decreased over years, reflecting the drug lag/loss in Japan. This was supported by the analysis of the time from the completion of GLP studies to submissions. Importantly, studies from China and Taiwan were emerging, reflecting the active drug development in China, which is not the adherent to the OECD Mutual Acceptance of Data (MAD). We also discuss the PMDA’s policies on the GLP studies conducted in non-MAD countries.

## 1 Introduction

Nonclinical safety studies conducted for the marketing authorization of medical products, such as drugs, medical devices, and regenerative medical products shall follow Good Laboratory Practice (GLP) in Japan. The Organisation for Economic Cooperation and Development (OECD) Mutual Acceptance of Data (MAD) is a framework that nonclinical safety studies conducted in test facilities which are successfully inspected by a competent GLP-compliance monitoring authority in an OECD country shall be accepted by other OECD countries including Japan (OECD, 1981). The framework has also opened to non-OECD member countries since 1997, and South Africa, Singapore, Argentina, Brazil, India, Malaysia, and Thailand currently adhere to the MAD so far. Under this framework, industries and governments could save at least 300 million euros annually (OECD, 2019). Based on the MAD framework, the Pharmaceuticals and Medical Devices Agency (PMDA) accepts GLP-compliant non-clinical studies (hereafter “GLP studies”) of medical products conducted in the test facilities successfully inspected by MAD adherent countries. On the other hand, PMDA conducts a product-based GLP inspection for studies conducted in non-MAD adherent countries. The product-based GLP inspection for individual marketing authorization aims to verify the GLP compliance status of specific studies submitted for the marketing authorization of medical products. Although the PMDA receives GLP studies from many countries, its geographical distribution and trend were not obvious. Such analysis could provide insights into drug development in Japan with respect to nonclinical testing.

Here, we analyzed the trends of GLP studies submitted to PMDA for the marketing authorization of pharmaceuticals and regenerative medical products from FY2017 to FY2023 with respect to the geographical origin of the GLP studies and the time from the completion of studies to the submission. US/Canada, Japan, Europe, and the UK were the major origins of GLP studies submitted to Japan, however, the share of studies conducted in Japan slightly decreased. This finding was also supported by the analysis of the time from the completion of GLP studies to submission. Our analysis also revealed that the GLP studies conducted in China and Taiwan are emerging these 3 years. This is consistent with the fact that China is a new originator of new pharmaceuticals. We discuss recent issues in drug development in Japan through the analyses. In response to the increased number of GLP studies conducted in China, we also describe PMDA’s policies on the nonclinical studies conducted in non-MAD adherent countries in this paper.

## 2 Trends of the GLP-compliant non-clinical studies submitted to the PMDA from FY2017 to FY2023

### 2.1 Origin of GLP-compliant non-clinical studies submitted to the PMDA

**FIGURE 1:**
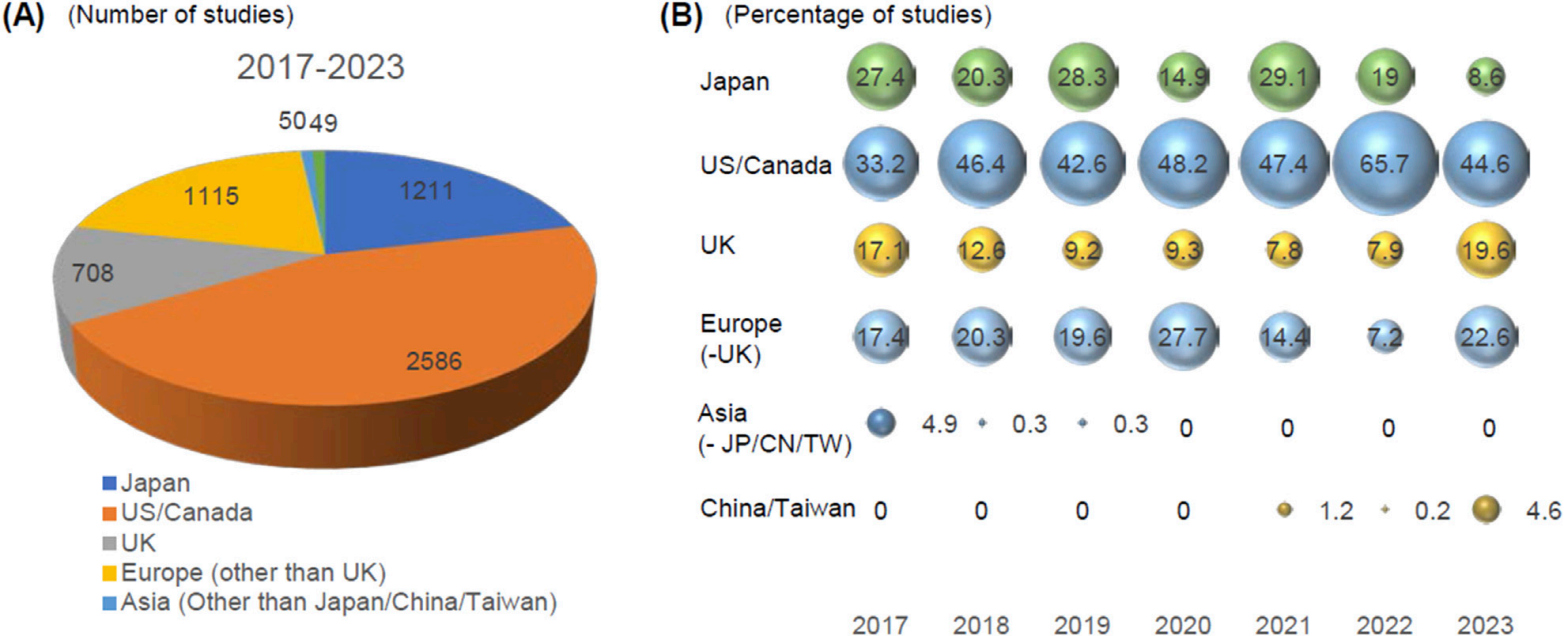
Analysis of the origin of GLP-compliant non-clinical studies submitted to PMDA. The location of test facilities where GLP studies were conducted was retrieved from the study reports (n = 5,473). **(A)** The origin of studies submitted from FY2017 to FY2023. The numbers of studies from each region are indicated. **(B)** The annual trends of the origin of GLP studies submitted to the PMDA. The percentage of the share of the studies are indicated in the figure. Asia other than JP/CN/TW: Asia other than Japan/China/Taiwan.

The key information of all GLP studies submitted to the PMDA has been consolidated in the database since FY2017, where 421 marketing authorization applications involving products with GLP studies were registered. Using the database, we analyzed 5,772 studies from the 421 applications submitted from FY2017 to FY2023 (mean, 13.7 studies/product, median, nine studies (1–80)/product). Of the 421 applications, 301 were concerned with new molecular entities (5,268 studies, median 14 (1–80) studies/product), and the rest of 120 were submitted for other purposes (504 studies, median nine studies (1–58) studies/product). To simplify the analysis, we excluded the phases of studies such as bioanalysis and histopathological examination accompanied with main studies. We retrieved the countries where the test facilities were located from each study report. We found that the US/Canada, Japan, Europe, and the UK were the major origins of GLP studies submitted to Japan (Figure 1A). US/Canada contribute most GLP studies submitted to PMDA. The percentage of GLP-compliant non-clinical studies from Europe decreased in 2021 and 2022 and recovered in 2023. Similar trend was observed for UK as well. It was not clear whether the change in trend for Europe and UK is meaningful. Asian countries other than China/Taiwan such as Korea and India had little contribution for the submitted GLP studies and recent 3 years, PMDA have not received data from this region. The analysis of annual trends also revealed that the percentage of studies conducted in Japan slightly decreased over the years (Figure 1B). This may reflect the reduced drug development activities in Japan, which is consistent with the recent decrease of the share of drugs from Japan (IQVIA Institiute for Human Data Science, 2022). Notably, GLP studies from China and Taiwan have been emerging in the last 3 years (Figure 1B). This is consistent with the worldwide development of new drugs originating from China (IQVIA Institiute for Human Data Science, 2022; Han et al., 2021; Ge et al., 2024), particularly oncology products, and PMDA that expects more GLP studies will be submitted for the marketing authorization of pharmaceuticals from this country (Ge et al., 2023).

### 2.2 Time from the completion of GLP-compliant non-clinical studies to submissions

**FIGURE 2:**
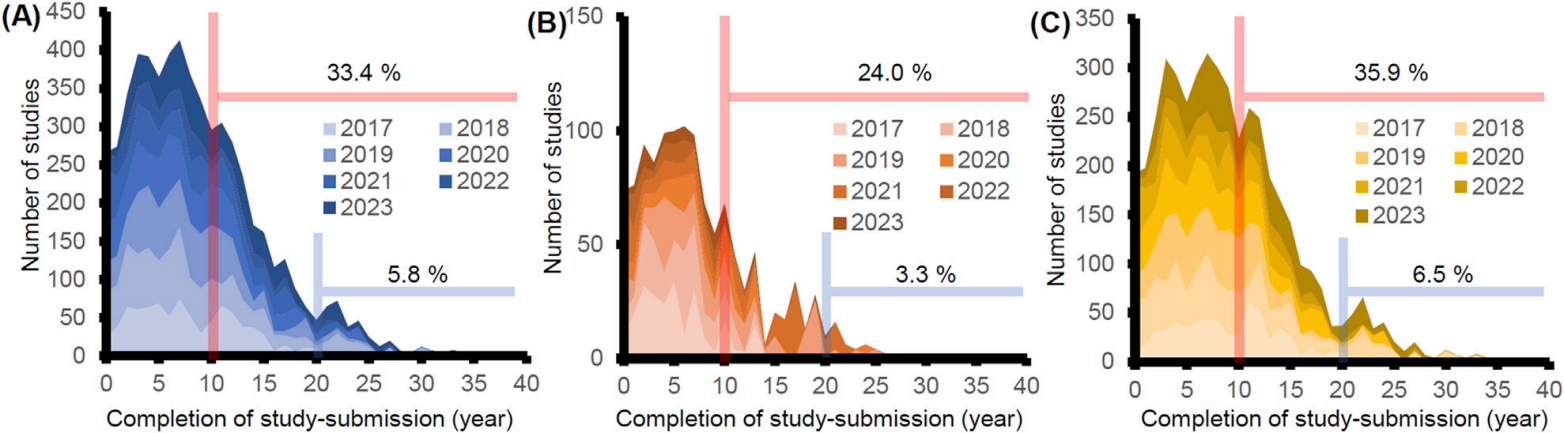
Analysis of the time from the completion of GLP-compliant non-clinical studies to the submission to PMDA. The years of the completion of GLP studies were retrieved from either the statements of compliance or those of quality assurance (n = 5,473). The years were calculated by subtracting the years of study completion from the years of submission. The percentages of studies submitted after >10 (red line) and 20 years (blue line) were calculated in each graph. **(A)** All studies were submitted from FY2017 to FY2023 (n = 5,473). **(B)** Studies conducted in Japan (n = 1,199). **(C)** Studies conducted outside Japan (n = 4,274).

Utilizing the same database, we also analyzed years needed for the submission of data to the PMDA from study completion. We calculated them by subtracting the completion year from the submission year to the PMDA. 33.4% and 5.8% of studies were submitted >10 and 20 years after study completion, respectively (Figure 2A). We compared the years needed for the submission for studies conducted in Japan and overseas. We found that 24.0% and 3.3% of the studies from Japan were submitted after 10 and 20 years, whereas 35.9% and 6.5% were submitted from overseas (Figures 2B,C). This difference is due to the delayed submission of the GLP studies conducted in oversea, which could partly be attributed to the submission lag of drugs in Japan reviewed in the recent paper (Enya et al., 2023). To further investigate if the difference stems from the delayed applications of the drugs to Japan, we examined the relationship between delayed submission of GLP studies and the lag time of the marketing approval of the products. Of the 65 applications submitted in FY2023, more than half of GLP-compliant non-clinical studies were submitted >10 years after the completion of studies in 19 products. Of the 19 products, nine products were submitted to the PMDA >5 years after the submission to either the US or EU. All GLP studies attached to them were conducted in overseas, and no GLP-compliant non-clinical studies were conducted in Japan. These findings also support the idea that submission lag is one of the reasons for the delayed submission of GLP studies from overseas.

## 3 Actionable recommendations

Our analysis of GLP studies submitted to the PMDA revealed the decreasing percentage of the studies conducted in Japan over the years, suggesting reduced drug development activities even in nonclinical development in Japan. To our best knowledge, this is the first systematic analysis of the GLP studies submitted to a competent receiving authority in terms of the location where a study was conducted and the time from study completion to submission. Similar analyses by other receiving authorities would give more insights into the trends of drug development worldwide. A gap in the number and timing of applications for marketing approval persists between Japan and US/EU, which is called drug lag/loss, and is a serious issue for patients with life-threatening diseases in Japan. Several causes of the drug lag/loss in Japan are discussed, including the difficulties in participating the multinational clinical trials (Shiga et al., 2025) or local requirement in Japan (Kobayashi and Narukawa, 2025). The less investments for the small-to mid-sized enterprises (SMEs) have led to the lack of the growth of SMEs and ultimately to the loss of the drug seeds in Japan (Ezell, 2022; Enya et al., 2025). This could also have resulted in reduced GLP studies conducted in Japanese test facilities The extra efforts by the government and industries should be required to maintain the drug development activities and tackle the drug lag/loss in Japan.

We also found active drug development in China, which was exemplified by the recent increase in the number of study submissions. The PMDA and other GLP compliance monitoring authorities of the MAD adherent countries assume that more GLP studies will be conducted in China and submitted to the MAD adherent countries. The number of Asian countries adhering to the MAD has been increasing over the years, but many Asian countries, including China, have not yet. In the framework of the OECD MAD, the MAD countries should accept data from other MAD countries, and the decision is legally binding (OECD, 1981). Conversely, the studies from non-MAD countries are not obligated to be accepted in MAD countries, and the decision of the acceptance of such data is the prerogative of each country. Notably, the same rule is applied to test facilities in non-MAD countries, even if the GLP compliance monitoring authorities in the MAD countries successfully inspected these facilities. The PMDA could accept data from test facilities in non-MAD countries only if the product-based inspection of the studies conducted by the PMDA is successful. The product-based inspection is conducted only after the application of the products, not before the application. Unlike a routine inspection which verifies GLP compliance of the test facilities and issues a GLP certificate valid for 3 years, no GLP certificate is issued for the product-based inspections and the studies successfully audited during the product-based inspection will not necessarily be accepted by other MAD countries. If the studies of concerns are judged to be not-in-compliance with GLP, applicants may need to repeat the studies as such studies will not be used for the dossiers for the marketing approval, leading to the delay in the marketing approval. Recently, the PMDA has modified the policy of the product-based inspection such that the product-based inspection could be conducted remotely only when the test facilities conducting the studies of concerns were successfully inspected by either the PMDA or the GLP compliance monitoring authorities of MAD adherent countries within 3 years. The PMDA’s policy regarding product-based inspection is available on the official website (https://www.pmda.go.jp/english/review-services/glp-gcp-gpsp/0002.html). The policy outlined here will be useful for those interested in performing early drug development and succeeding drugs under development from non-MAD countries.

## 4 Conclusion

Our analysis of GLP studies submitted to the PMDA for the application reflected the drug lag/loss in Japan. The activities to support the innovation and pre-clinical studies are essential to develop sustainable ecosystem of the drug development. Given that some global pharmaceutical companies acquire the licenses from Chinese pharmaceutical companies, they would need extra care for studies conducted in non-MAD countries even if the test facilities conducting these studies are certified by the GLP compliance monitoring authority of MAD adherent countries. Our paper provided clear policies on non-MAD studies in the marketing authorization of pharmaceuticals in Japan and they will serve as a reference for pharmaceutical companies in early drug development.
